# Feasibility and Effectiveness of Using Wearable Activity Trackers in Youth: A Systematic Review

**DOI:** 10.2196/mhealth.6540

**Published:** 2016-11-23

**Authors:** Nicola D Ridgers, Melitta A McNarry, Kelly A Mackintosh

**Affiliations:** ^1^ Deakin University, Geelong, Australia Institute for Physical Activity and Nutrition (IPAN) School of Exercise and Nutrition Sciences; ^2^ Applied Sports Science Technology and Medicine Research Centre (A-STEM) College of Engineering Swansea University Swansea United Kingdom

**Keywords:** behaviour change, electronic activity monitor, mHealth, physical activity

## Abstract

**Background:**

The proliferation and popularity of wearable activity trackers (eg, Fitbit, Jawbone, Misfit) may present an opportunity to integrate such technology into physical activity interventions. While several systematic reviews have reported intervention effects of using wearable activity trackers on adults’ physical activity levels, none to date have focused specifically on children and adolescents.

**Objective:**

The aim of this review was to examine the effectiveness of wearable activity trackers as a tool for increasing children’s and adolescents’ physical activity levels. We also examined the feasibility of using such technology in younger populations (age range 5-19 years).

**Methods:**

We conducted a systematic search of 5 electronic databases, reference lists, and personal archives to identify articles published up until August 2016 that met the inclusion criteria. Articles were included if they (1) specifically examined the use of a wearable device within an intervention or a feasibility study; (2) included participants aged 5-19 years old; (3) had a measure of physical activity as an outcome variable for intervention studies; (4) reported process data concerning the feasibility of the device in feasibility studies; and (5) were published in English. Data were analyzed in August 2016.

**Results:**

In total, we identified and analyzed 5 studies (3 intervention, 2 feasibility). Intervention delivery ranged from 19 days to 3 months, with only 1 study using a randomized controlled trial design. Wearable activity trackers were typically combined with other intervention approaches such as goal setting and researcher feedback. While intervention effects were generally positive, the reported differences were largely nonsignificant. The feasibility studies indicated that monitor comfort and design and feedback features were important factors to children and adolescents.

**Conclusions:**

There is a paucity of research concerning the effectiveness and feasibility of wearable activity trackers as a tool for increasing children’s and adolescents’ physical activity levels. While there are some preliminary data to suggest these devices may have the potential to increase activity levels through self-monitoring and goal setting in the short term, more research is needed to establish longer-term effects on behavior.

## Introduction

Physical inactivity is a global pandemic and has been identified as the fourth leading cause of death worldwide [1]. Regular physical activity plays a critical role in preventing precursors to metabolic and cardiovascular ill health in children [2], providing numerous health benefits during childhood that persist into adulthood [3]. Such health benefits include protective effects on bone health, as well as positive effects on fitness, body fat, and blood pressure [4]. Several countries (eg, the United States, United Kingdom, and Australia) recommend that children should engage in at least 60 minutes of moderate- to vigorous-intensity physical activity (MVPA) every day to benefit health [5-7]. However, the majority of children and adolescents (defined as youth hereinafter) do not meet these recommended levels and are therefore not sufficiently active to accrue the associated health benefits [8-10]. Since physical inactivity is a major, yet modifiable, risk factor for the burden of disease, there is a need for effective, preventive interventions that aim to increase physical activity levels in this population.

Self-monitoring has been identified as an effective behavior change technique that has been used in behavioral interventions targeting increases in physical activity levels [11]. Indeed, self-monitoring and feedback are fundamental to increasing awareness of individual physical activity levels, which is particularly important given that youth are unlikely to change their behavior if they do not know how active they actually are and how this translates to government guidelines. Specifically, Corder and colleagues found that approximately 60% of inactive adolescents thought that they met physical activity guidelines [12], suggesting that they may see no need to change their behavior, despite the associated health benefits. Traditionally, hip-worn pedometers have been used to increase individuals’ awareness of their physical activity [13]; however, participants are required to record their activity at the end of each day, which can be burdensome for them [13]. In recent years, there has been increasing interest in emerging technologies and wearable sensors as self-monitoring tools for promoting physical activity levels [14]. The proliferation of wearable activity trackers, as well as their growing commercial availability, popularity, and widespread adoption [15], presents an opportunity to integrate such technologies into physical activity interventions. While ownership data are not available for youth, it is estimated that 10% and 20% of US and Australian adults, respectively, own some form of wearable technology [15,16]. An integral component of wearable activity trackers, such as Fitbit and Jawbone, is the automation of real-time physical activity tracking [17]. The wireless syncing of such devices to Web- or app-based profiles not only negates the burden of manual data entry, but also enables the wearer to self-monitor against physical activity recommendations or set goals [14,17].

To date, physical activity research has primarily focused on establishing the validity and reliability of wearable activity trackers for measuring a range of outcomes, including steps and sleep [18]. In comparison, little is known about the feasibility and effectiveness of these devices as a tool for increasing physical activity levels, whether in isolation or in combination with other strategies. A recent review reported that there was some initial evidence that wearable activity trackers can increase physical activity levels, though only studies conducted in adult populations were included [19]. Given that engagement with technology is a highly valued behavior for youth and plays an important role in different domains of their lives (eg, education, socialization, and entertainment; [20]), there is a need to establish whether wearable activity trackers are feasible and effective in changing physical activity levels in this population. Such information is important for informing future physical activity interventions and has the potential to contribute to the development of public health guidance concerning the role of these tools in physical activity and health promotion practice.

The aim of this review, therefore, was to examine the effectiveness of wearable activity trackers as a tool for increasing children’s and adolescents’ physical activity levels. We also examined the feasibility of using such technology in youth populations (defined as those in the age range 5-19 years).

## Methods

We conducted a systematic literature search in accordance with the Preferred Reporting Items for Systematic Reviews and Meta-Analyses (PRISMA) statement [21]. We searched 5 electronic databases (PubMed, Web of Science, SPORTDiscus, Scopus, and ProQuest Central). Search strategies for the different databases included the following search strings in four main areas: wearable activity trackers (electronic track*, electronic activ*, electronic monitor, electronic fitness track*, wearable device, wearable act*, wearable track*, consumer wearable, Fitbit, SenseWear, Jawbone, Nike Fuelband, PAM), population (child*, adolescent, youth), study design (intervention, trial, feasibility), and outcome variable (physical activity, energy expenditure, fitness, exercise). The full search strategies for each database are presented in Multimedia Appendix 1. Articles that had been published in peer-reviewed journals or conference proceedings were considered for review. We did not include abstracts, dissertations, systematic reviews, and case studies. In addition to electronic searches, we also searched our personal collections and the bibliographies of retrieved studies. This is a commonly used approach for the identification of additional relevant studies for potential inclusion in systematic reviews [22].

For the purpose of this review, we defined wearable activity trackers as an electronic device with the following features: was designed to be worn on the user’s body; uses accelerometers, altimeters, or other sensors to track the wearer’s movements or biometric data, or both; and can provide feedback, beyond the display of basic activity count information, via the monitor display or through a partnering app to elicit continual self-monitoring of activity behavior [19,23]. To be included in the review, studies were required to (1) specifically examine the use of a wearable device within an intervention or a feasibility study; (2) include participants aged 5-19 years old; (3) have a measure of physical activity as an outcome variable for intervention studies; (4) report process data concerning the feasibility of the device in feasibility studies; (5) be published between the start date of each electronic database and August 2016; and (6) be published in English. We excluded studies that reported study protocols, used mobile phones rather than a wearable activity tracker, used mobile phone or tablet apps without an accompanying wearable activity tracker, or only used the wearable activity tracker to evaluate an intervention (eg, worn at baseline and postintervention). In the event that a wearable activity tracker was used in conjunction with other tools, such as Facebook to share activity tips, studies were eligible for inclusion if physical activity data were reported or the feasibility of the device was reported separately. Studies that implemented the use of such technologies in clinical populations were eligible for inclusion if the focus was on using a wearable device to increase physical activity levels. When studies were still in press or were an advanced publication ahead of print but had a unique digital object identifier, they were eligible for inclusion. Conference proceedings were eligible for review due to the potential for such devices to be examined in different disciplines (eg, computer science) where such outputs are often more reputable than journal articles.

All authors independently assessed the results obtained from the initial literature search. Articles were screened in 4 steps: first, we removed duplicates, then screened the title, abstract, and full text. We then screened the articles based on the inclusion and exclusion criteria outlined above. If we could not determine suitability during the screening of the title and abstract, we accessed full-text articles and compared them against the inclusion criteria. Any disagreements were settled by discussion between the authors. We then extracted the following data using a standardized form for each study that met the inclusion criteria: country of study, study design, sample characteristics (eg, sample size, age), wearable device used, and results. The first author extracted the data, which were checked by the remaining authors (MAM, KAM). We then undertook a narrative review of the included studies.

The second and last authors (MAM, KAM) independently assessed the risk of bias in the intervention studies that met the inclusion criteria. We adapted the criteria for assessing risk of bias from the *Methods Guide for Comparative Effectiveness Reviews* [24] and previous reviews in similar areas [19]. We identified 8 criteria as being important to this review: (1) participants were allocated randomly; (2) an adequate proportion of participants had complete data for the outcome variable (ie, no more than 20% of data were missing); (3) data were analyzed according to group allocated; (4) the study population was representative of the population of interest; (5) the timing of outcome assessments was similar in all groups; (6) the study reported the validity of the device used (either data were provided in the article or there was an appropriate reference to the original study); (7) the study reported the reliability of the device used (either data were provided in the article or there was an appropriate reference to the original study); and (8) the study was conducted independently of the manufacturer of the device used. We assessed only the last 3 risk-of-bias criteria for feasibility studies given the nature of such study designs. Each criterion was scored as “yes” (1), “no” (0), or “unsure” (?).

## Results

**Figure 1:**
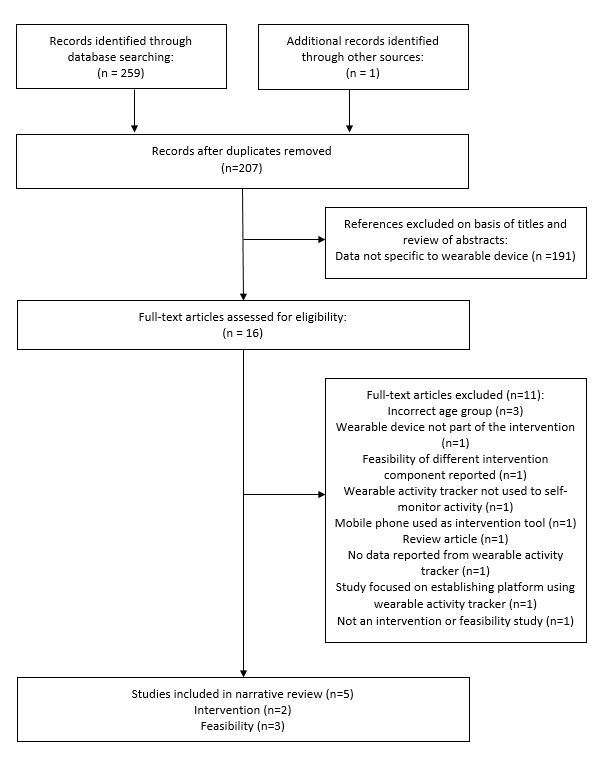
Flow diagram of screening process and results.

**Figure 2:**
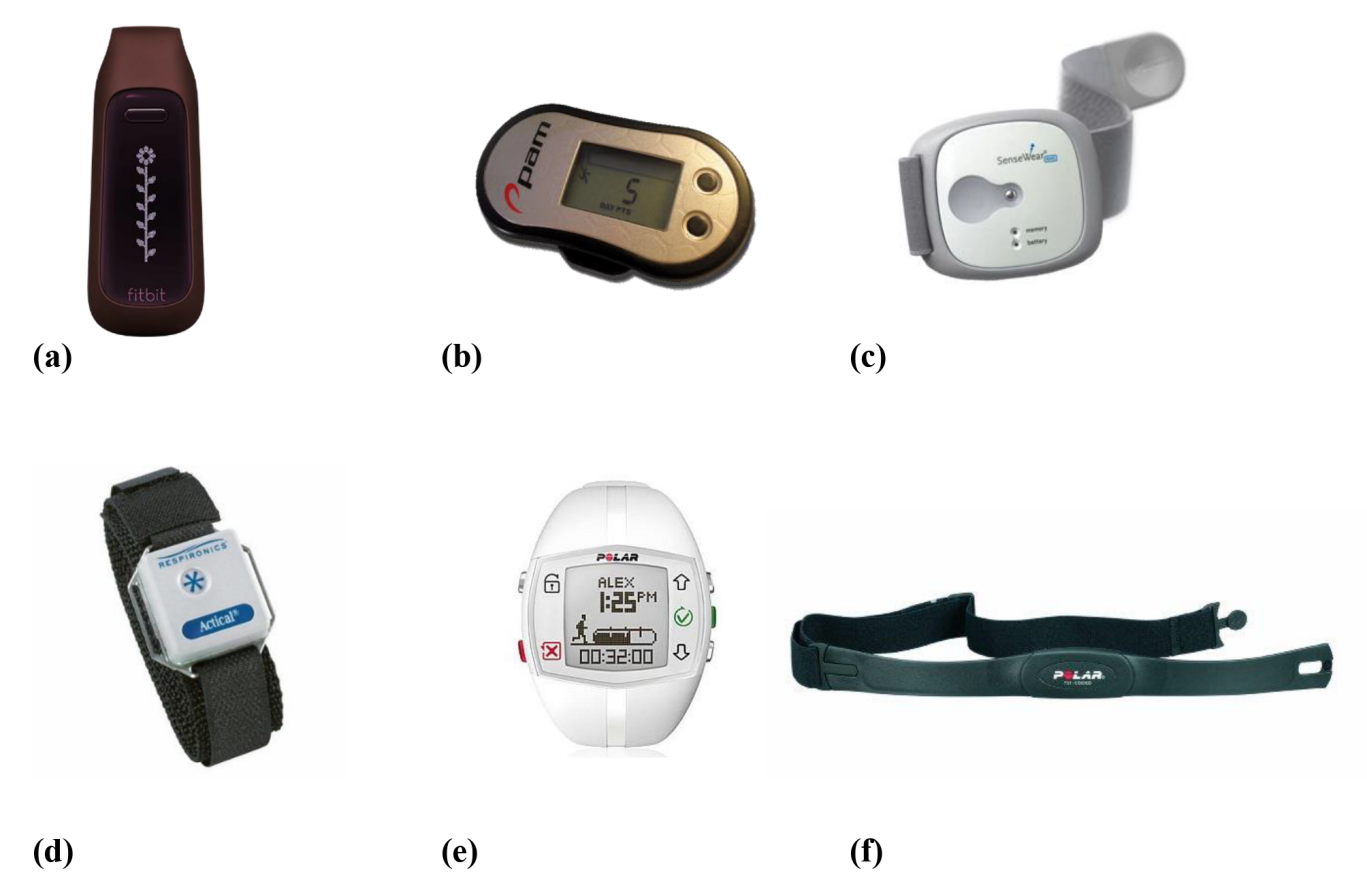
Wearable devices used in the included studies: (a) Fitbit One, (b) the PAM (new model shown), (c) SenseWear, (d) Actical, (e) Polar Active, and (f) Polar heart rate monitor.

We screened and analyzed data in August 2016. Through the systematic search, we initially identified 259 articles, then identified 1 additional article through other sources. Of these, 5 were included in the review (Figure 1): 3 were intervention studies [25-27] and focused primarily on increasing physical activity levels across the whole day [26,27] or during recess [25], and 2 studies were classed as feasibility studies [28,29]. Of the intervention studies, 2 [26,27] also included process measures about the device used. The majority of the studies (n=4) focused on children and were conducted in the United States [25,27-29]. The main brand of wearable device used was Fitbit [25,27,28]. Table 1 reports the characteristics of each included study. Figure 2 shows the different wearable devices used in the included studies, and Table 2 provides an overview of the features of these devices.

Table 3 reports the risk of bias for each study. All were conducted independently of the manufacturers of the device(s) used, while 2 studies reported the reliability and validity of the devices used. Of the intervention studies conducted, only 1 used a randomized controlled trial design [26].

**Table 1:** Summary of included studies on the effectiveness and feasibility of wearable activity trackers in youth (chronological order by study design).

Study	Country	Participants	Type of study	Study design and description	Device examined	Outcomes assessed
Slootmaker, 2010 [26]	Netherlands	Adolescents (13-17 years old), 32 boys, 55 girls (15.1 years) at baseline	Intervention plus feasibility component	Least active group of youth recruited. Randomized to intervention or control. 3-month Web-based intervention combining self-monitoring, goal setting, device and PAM COACH.	PAM and PAM COACH	Physical activity: Activity Questionnaire for Adolescents and Adults (self-report). Time spent in SED^a^, LPA^b^, MPA^c^, and VPA^d^. Process measures: evaluation of PAM and PAM coach.
Hayes, 2015 [25]	USA	6 grade-3 girls (aged 8 years old) from 1 school; intact social group	Intervention	Recess intervention (22 sessions in total). Fitbit used to self-monitor physical activity levels against set goals. Tangible rewards provided if goals met.	Fitbit (model not reported)	Steps/recess. MVPA^e^ (min) during recess.
Hooke, 2016 [27]	USA	16 children (5 boys, 11 girls) aged mean 8.7, SD 3.1 years; participants receiving a cycle of maintenance chemotherapy for lymphoblastic leukemia	Intervention plus feasibility component	Used for 17 days before and 5 days after a corticosteroid pulse. Step goal tailored based on data and daily feedback against goal provided (either to increase or maintain physical activity). Goal set in Fitbit website by study nurse for participants to track progress.	Fitbit One	Steps/day. Feasibility component included ease of recruitment, ease of use and enjoyment of Fitbit, and days of wear.
Schaefer, 2014 [29]	USA	24 children (11 boys, 13 girls) aged 7-10 years (mean 8.9, SD 1.3 years)	Feasibility	Each child wore a different monitor for 1 week (4 weeks total). Underwent structured interview about each device and then summary (exit) interview at the end, with child and parents interviewed separately.	Actical SenseWear Polar Active Polar heart rate monitor	Frequency of removal, reasons for removal, enjoyment, comfort of use, favorite/least favorite device characteristics. Devices also ranked in terms of most and least favorite.
Schaefer, 2016 [28]	USA	34 children (22 boys, 12 girls) 11-12 years old (mean age 12.6 years); attending a low-socioeconomic-status school	Feasibility	6-month feasibility study. Initially asked to wear devices during after-school program, which then increased to daily wear.	Fitbit One	Fitbit data (ie, steps). Interviews examining experiences of using the Fitbit.

^a^SED: sedentary time.

^b^LPA: light-intensity physical activity.

^c^MPA: moderate-intensity physical activity.

^d^VPA: vigorous-intensity physical activity.

^e^MVPA: moderate- to vigorous-intensity physical activity.

**Table 2:** Overview of features of wearable devices used in the included studies on the effectiveness and feasibility of wearable activity trackers in youth.

Device	Location worn	Main measures	Device display	Compatibility	Sensors	Memory	Waterproof
Fitbit One	Waist	Steps, stairs, distance, calories, sleep	Yes	Personal computer, iOS, Android, Windows	Accelerometer (3 axis), altimeter	Up to 23 days	No
PAM	Waist	Physical activity score	Yes	Personal computer	Accelerometer (3 axis)	Not reported	No
SenseWear	Upper arm	Physical activity, energy expenditure, steps, sleep	No (optional display required)	Personal computer	Accelerometer (3 axis), heat flux, galvanic skin response, skin temperature, near-body ambient temperature	Up to 34 days	No
Actical	Wrist, waist, ankle	Physical activity, energy expenditure, steps	No	Personal computer	Accelerometer (omnidirectional)	Up to 194 days	Yes
Polar Active	Wrist	Physical activity, steps, calories, sleep	Yes	Personal computer, iOS, Android	Accelerometer (3 axis)	21 days (activity diary)	Yes
Polar heart rate monitor	Chest	Heart rate, calories	No	Personal computer	Heart rate	Up to 30 hours	Yes

**Table 3:** Risk-of-bias results^a^ in studies on the effectiveness and feasibility of wearable activity trackers in youth.

Study	Random allocation	Minimal missing data	Analyzed in group allocated	Representative sampling	Timing of outcome assessments	Reliability of device	Validity of device	Independence from device manufacturer
Hayes, 2015 [25]	0	0	1	0	1	1	1	1
Hooke, 2016 [27]	0	1	1	0	1	1	1	1
Schaefer, 2014 [29]	N/A^b^	N/A	N/A	N/A	N/A	0	0	1
Schaefer, 2016 [28]	N/A	N/A	N/A	N/A	N/A	0	0	1
Slootmaker, 2010 [26]	1	1	1	1	1	0	0	1

^a^Scored as follows: 1 = yes; 0 = no.

^b^N/A: not applicable.

### Intervention Studies

In the Netherlands, Slootmaker and colleagues [26] investigated the effectiveness of the PAM monitor in combination with PAM COACH for increasing the physical activity levels of adolescents (aged 13-17 years; n=87). Activity data from the PAM device were uploaded to PAM COACH, which was a Web-based system for self-monitoring activity levels and setting goals. At the conclusion of the 3-month intervention, girls in the intervention group increased their self-reported weekly moderate physical activity relative to controls (equated to ~59 min/day), though this was not evident at 8 months postintervention. No intervention effects were observed for boys after 3 months, though self-reported sedentary time was lower at 8 months relative to boys in the control group (~257 min/day). Greater adherence to the study (eg, log-in and upload frequency to PAM COACH) was not associated with greater physical activity. Minimal attrition was observed, with 78% and 91% of participants providing follow-up data at 3 and 8 months postintervention, respectively. Overall, the PAM was viewed positively by participants, and 65% reported regular wear, though monitor loss (12%) and damage (7%) may have influenced the results.

Hayes and Van Camp [25] used the Fitbit (model not reported) as a tool for increasing 6 grade 3 (8 years old) girls’ physical activity levels during school recess. After baseline data were collected from 7 recess periods, girls were provided with step goals (increments based on baseline data) for 7 further recess periods and were encouraged to self-monitor their steps against these goals. Following this, data were then collected for a further 7 periods (no step goals provided). The project culminated in a final intervention session where 3 goals were given, and a tangible reward (eg, a small toy) was provided based on the goal(s) achieved. The number of steps taken increased by 47% from baseline (1326 steps) to intervention (1956 steps; contribution of 18% to daily step recommendations [30]), while the proportion of time spent in MVPA increased from 4% to 25%, which equates to 5 minutes of MVPA during recess, or a contribution of 8% to daily recommendations [5]. Without the use of the Fitbit to self-monitor recess activity, steps taken and MVPA decreased to initial baseline levels. Basic process evaluation measures suggested that data were lost due to syncing issues, particularly during later recess sessions.

Hooke and colleagues [27] examined the efficacy of the Fitbit One to promote physical activity in clinical settings. A total of 16 children (mean age 8.7, SD 3.1 years) with acute lymphoblastic leukemia were provided with a Fitbit to wear for 17 days prior to and 5 days during a corticosteroid pulse. Monitoring over 3 days was used to identify baseline activity levels. Step goals were then tailored for each participant by a research nurse, and daily feedback was provided against these goals. No significant increases in daily steps were recorded, though there was a trend for steps to increase from week 1 to week 2 (average of 269 steps/day; 2% of daily step guidelines [30]) but to decrease from week 2 to week 3 (average of 307 steps/day). Process evaluation indicated that participants and their families were able to use the Fitbit One and the accompanying website, that they liked the Fitbit, and that data were available for 92% of measured days.

### Feasibility Studies

Schaefer and colleagues conducted 2 feasibility studies in US primary school-age children [28,29]. In the first, 24 children each wore 4 activity monitors (Actical, SenseWear, Polar Active, and Polar heart rate monitor) separately for 1 week [29]. Of these activity monitors, the Polar Active and SenseWear met the definition of a wearable activity tracker. Following each week of wear, children and their parents were interviewed about their experiences of using the monitors. Overall, the Polar Active was the most popular monitor, with its comfort and feedback features (including a clock function) noted. It was used for 98% of the total time. In comparison, the SenseWear was the least popular, largely due to its placement on the arm (uncomfortable, embarrassing). This also corresponded with its lack of use (28% of the total time). No reactivity to wear was reported for the Polar Active.

In their second study, Schaefer and colleagues examined the feasibility of the Fitbit One in children aged 11-12 years attending a school located in an area of socioeconomic disadvantage [28]. Initially, 24 children were provided with a Fitbit to monitor their activity levels during an after-school program. After several weeks (approximately 1 month), children were provided with a Fitbit to wear every day for 5 months. On average, children accumulated 8406 steps/day. The number of steps taken increased during the monitoring phase, but not significantly. On average, 58 days of data were collected from each participant during the study, of which 19 days were considered to be valid days (overall use of 15%). Only 2 participants were still using their Fitbit at the end of the study (8%). Interview data indicated that, while the range of functions were well received, the design of the Fitbit One was unpopular and it was easy to forget to wear. Some children reported using the monitor to try to change behavior, while others (mainly boys) used it to compete against each other. One of the biggest barriers to use was the children’s ability to sync and access their data outside of the after-school program.

## Discussion

### Principal Findings

This systematic, narrative review evaluated the effectiveness of wearable activity trackers as a tool for increasing children’s and adolescents’ physical activity levels. We also examined the feasibility of using wearable activity trackers in this population. Overall, there is a dearth of studies that have reported the use of wearable activity trackers in youth populations to increase activity levels. We identified 3 intervention studies, with 1 implemented in school-age children [25], 1 in a clinical population [27], and 1 with adolescents [26], the latter being the only study to use a randomized controlled trial design. There was some evidence to suggest that wearable activity trackers may have the potential to increase youth activity levels, with increases in physical activity compared with baseline or a control group observed. In addition, there was some evidence that wearable activity trackers were viewed positively by youth and they enjoyed wearing them. However, given that the studies had numerous methodological shortcomings and the majority of the reported differences were largely nonsignificant, it is clear that further research using rigorous and well-designed methodologies is needed to establish the effectiveness of these devices for increasing youth activity levels.

### Intervention Effects

The limited intervention effects observed in studies included in this review may be attributable to several factors. First, the length of the interventions implemented ranged from 19 days to 3 months. It is possible that the shorter intervention periods were not sufficient to change behavior, a notion supported by a recent review that highlighted that behavioral interventions of longer durations (≥6 months) had greater success in changing physical activity levels [31]. There is a clear need for studies using longer measurement periods to examine the effectiveness of these devices in youth. Second, it is not clear whether the interventions were grounded on behavioral theories, which are critical for intervention effectiveness [32]. Third, 2 of the studies may have been underpowered to detect a change in physical activity due to the smaller number of children recruited [25,27]. Fourth, intervention effects were not examined using validated objective monitors (eg, accelerometers). While several studies used data from the wearable activity tracker, these devices have not been validated for assessing physical activity outcomes in youth to date [18]. This could be viewed as a limitation of these included studies. However, this may be less of an issue if the focus of an intervention study is to use the device as a tool to facilitate behavior change rather than to evaluate the outcome (ie, validity and reliability should be established prior to such use). In the only study to report significant effects, self-reported physical activity data were collected using a questionnaire with low validity [26]. There is a clear need for longer-term studies using randomized controlled trials that are grounded on behavioral theories to identify the effectiveness of wearable activity trackers for changing youth physical activity behavior.

### Self-Monitoring Using Wearable Activity Trackers

A common feature of the identified intervention studies was that the wearable activity tracker was used to self-monitor physical activity in combination with different intervention approaches [17]. Unsurprisingly, these intervention approaches largely consisted of goal setting, identified as an effective behavior change technique [11] to enhance physical activity levels [33]. However, there was some variability in who set the goals. In the interventions with children, the researchers set the goals based on baseline values obtained, and then provided regular support [27] or rewards [25] in relation to reaching the goals. In contrast, the adolescents had an opportunity to set their own activity goal and then received tailored advice to achieve these based on their preferred activities [26]. While research has shown that assigned goals are as effective as self-set goals, provided the reason for the goal is given [34], it is unclear whether the included studies provided this information to participants. It is important for future research to establish how youth engage with wearable activity trackers (eg, is the frequency of self-monitoring mediated by the activity goal source?), as this will provide critical insights into how these devices can be integrated into future interventions and strategies for engaging children and adolescents in the behavior change process.

### Sustainability of Wearable Activity Trackers Over Time

Arguably, one of the biggest concerns regarding wearable activity trackers is whether individuals sustain their engagement with the technology over time [16]. Research has suggested that approximately one-third of US adults stop using their wearable activity tracker after 6 months [16], with expectation mismatch (ie, the technology doesn’t do what was expected) a commonly cited reason [35]. While it is difficult to draw any conclusions about this due to the small number of studies identified and the variability in the length of time the wearable activity trackers were worn, there is some preliminary evidence to suggest that youth might regularly use the technology to self-monitor their physical activity levels when the technology is integrated into an intervention [26,27], but sustained use may not be observed in the longer term when these devices are simply provided to youth to use [28]. Interestingly, Slootmaker and colleagues found that the frequency that adolescents uploaded their data to PAM COACH was not associated with physical activity, yet physical activity levels were found to increase in girls at 3 months and sedentary time decreased in boys at 8 months [26]. These results could be explained by the lower activity levels of the girls in the study compared with boys; therefore, girls could achieve greater gains in activity levels [26]. However, it is also possible that there are different degrees of engagement, ranging from brief glances at the device [36], to tracking activity across the day using the monitor, to using specific functions within the accompanying app (eg, trends data), which may mediate the efficacy of the device on activity levels. Research is needed to provide further evidence about how youth engage with these devices and the accompanying apps over time (eg, attrition rates), whether this differs by population subgroups (eg, sex, age), whether the engagement with different features may have differential effects, and reasons for potential changes in use over time. Such research will be critical for identifying how to incorporate wearable activity trackers into interventions, and for informing best practice in future physical activity interventions and health promotion practice.

### Feasibility of Wearable Activity Trackers in Youth

This review identified few studies that have examined the feasibility of using wearable activity trackers in children and adolescents, either as part of an intervention or as a stand-alone study. While these technologies offer significant promise for increasing physical activity levels and benefiting health promotion practice [14] or, potentially, clinically relevant outcomes in healthy and clinical populations [27], it is important to establish whether wearable activity trackers are a practical tool for youth to use regardless of the setting. Overall, the results from this review suggest that such devices were viewed positively by youth and their parents [26-29], and that they appreciated the devices’ range of functions, which included the tracking of physical activity. Ease of use, comfort, and aesthetics were important to the participants [28,29], which is an interesting point to note given that such devices are unlikely to have been developed with youth in mind [35]. Such factors have been previously identified as important and potential barriers to use in adults [37,38]. Interestingly, while there was some evidence that youth used their wearable activity tracker to compete with (boys) or support (girls) each other [28], which has also been observed in adults [37], few studies noted concerns over the accuracy of the devices. Schaefer and colleagues found that, while some children did test the accuracy of the monitors [28], this did not appear to influence use. Of potentially greater concern, access to technology was identified as a potential issue in low-socioeconomic-status youth, such as the ability to sync and access data at home [28]. This supports a previous study that found that the use of a mobile phone app that enabled boys from low-socioeconomic areas to track their goals and behavior was moderate, due to their prioritizing data for entertainment over the app [39]. As such, we recommend that researchers examine the feasibility and acceptability of different devices, and we suggest that youth are interested in using wearable activity trackers and that these devices are feasible for use in both clinical and healthy populations. However, it must be noted that there are issues concerning the age required (≥13 years old) to hold an account associated with wearable activity trackers that may preclude their use with children, unless alternative feedback mechanisms are employed (eg, researcher-led feedback [25,27]). Identifying how best to integrate the functions and features of wearable activity trackers into physical activity interventions, in order to maximize their potential within the population of interest, should be a future area of research.

### Limitations

There are some limitations in this review that warrant attention. First, this review highlights the surprisingly small number of studies that have used wearable activity trackers in youth despite their widespread prevalence in daily life. This makes it difficult to establish any firm conclusions. Given the pervasiveness of these technologies and widespread appeal, further research is needed to explore these issues in youth in order to inform interventions and public health guidance. In addition, research is needed to establish whether differential effects are observed based on the age of the participants. Second, the quality of the included studies was low, with only 1 study using a randomized controlled design and evaluating intervention effects over a 3-month period. There is a need for more rigorous and robust intervention designs to evaluate the longer-term effectiveness of these devices on youth physical activity levels. Third, while a range of monitors were used in the included studies, some monitors have since been discontinued (eg, PAM, SenseWear) or largely usurped by new models (eg, Fitbit). In addition, the validity and reliability of these devices in youth has not been established.

### Conclusions

There is a paucity of literature concerning the effectiveness of wearable activity trackers as a tool for increasing children’s and adolescents’ physical activity levels. Additionally, little research has documented the feasibility of such technology in youth. While there are some preliminary data to suggest that wearable activity trackers are feasible and may have the potential to increase physical activity levels through self-monitoring and goal setting in youth, there is a clear need for more research to examine these issues in youth using robust studies with longer measurement periods. Given the constant changes in the wearable technology market (eg, newer devices and models are regularly available), research should primarily focus on the use of the device as a tool for changing behavior in youth in interventions. Focusing on features common to different wearable device brands (eg, self-monitoring displays, accompanying apps, and biofeedback features) may be important for establishing the feasibility of these technologies in youth rather than that of the individual monitors per se. Based on this review, feasibility research should establish how youth engage with this technology, whether adherence and engagement are sustained or change over time, and whether effects vary based on age and sex. Such information will inform the development of future interventions and identify how to maximize the potential contribution that such pervasive technologies could make to physical activity promotion in youth.

## Appendix

Multimedia Appendix 1 Search strategy for each database.

## Abbreviations

MVPA: moderate- to vigorous-intensity physical activity
PRISMA: Preferred Reporting Items for Systematic Reviews and Meta-Analyses
