# Supplementary material for: Feasibility and Effectiveness of Using Wearable Activity Trackers in Youth: A Systematic Review
Source: JMIR Mhealth Uhealth. 2016 Nov 23;4(4):e129. doi: 10.2196/mhealth.6540 (PMC5143467; doi:10.2196/mhealth.6540)
Supplement: Multimedia Appendix 1 [file mhealth_v4i4e129_app1.pdf]

**Appendix 1: Search strategy for each database**

| Database | Search String | Results |
|---|---|---|
| PubMed | (((((electronic track* OR electronic activ* AND track* OR electronic activ* AND monitor* OR electronic fitness track* OR wearable device OR wearable act* AND track* OR consumer wearable OR Fitbit OR Jawbone OR Nike Fuelband OR SenseWear OR PAM))) AND ((Intervention OR trial OR feasibility))) AND ((Child* OR Adolescent* OR youth))) AND ((Physical act* OR Exerc* OR Fitness OR Energy expenditure)) | 33 |
| Scopus | (electronic track* OR electronic activ* AND track* OR electronic activ* AND monitor* OR electronic fitness track* OR wearable device OR wearable act* AND track* OR consumer wearable OR Fitbit OR Jawbone OR Nike Fuelband OR SenseWear OR PAM) AND (Intervention OR trial OR feasibility) AND (Child* OR Adolescent* OR youth) AND (Physical act* OR Exerc* OR Fitness OR Energy expenditure) | 10 |
| ProQuest Central | all(electronic track* OR electronic activ* AND track* OR electronic activ* AND monitor* OR electronic fitness track* OR wearable device OR wearable act* AND track* OR consumer wearable OR fitbit OR Jawbone OR Nike fuelband OR SenseWear OR PAM) AND all(Intervention OR trial **OR feasibility**) AND all(Child* OR Adolescent* OR youth) AND all(Physical act* OR Exerc* OR Fitness OR Energy expenditure) | 114 |
| Web of Science | TS=(electronic track* OR electronic activ* AND track* OR electronic activ* AND monitor* OR electronic fitness track* OR wearable device OR wearable act* AND track* OR consumer wearable OR fitbit OR Jawbone OR Nike fuelband OR Sensewear OR PAM) AND TS=(Intervention OR trial OR feasibility) AND TS=(Child* OR Adolescent* OR youth) AND TS=(Physical act* OR Exerc* OR Fitness OR Energy expenditure) | 94 |
| SPORTDiscus | AB ( (electronic track* OR electronic activ* AND track* OR electronic activ* AND monitor* OR electronic fitness track* OR wearable device OR wearable act* AND track* OR consumer wearable OR fitbit OR Jawbone OR Nike fuelband OR Sensewear OR PAM) ) AND AB ( (Intervention OR trial OR feasibility) ) AND AB ( (Child* OR Adolescent* OR youth) ) AND ( (Physical act* OR Exerc* OR Fitness OR Energy expenditure) ) | 8 |
